# Rhabdomyomatous mesenchymal hamartoma presenting as a big subcutaneous mass on the neck: a case report

**DOI:** 10.1186/1752-1947-8-410

**Published:** 2014-12-06

**Authors:** YuLan Wang, HaiHua Zhao, XinHua Yue, XinPing Tang, Li Ma, Yong Fu, PeiYi Zhang

**Affiliations:** Department of Pathology, Uromuqi General Hospital, LanZhou, Military District, 830054 China

**Keywords:** Immunohistochemistry, Neck, Rhabdomyomatous mesenchymal hamartoma

## Abstract

**Introduction:**

We describe the location, size, histopathologic aspect and immunohistochemical expression of a rhabdomyomatous mesenchymal hamartoma, with the aim of providing useful information for its correct diagnosis.

**Case presentation:**

A 31-year-old Chinese man first presented 2 years previously with a solitary subcutaneous mass on the left side of his neck and under his mastoid process; the mass’s size was 2×2cm. The mass increased in the size in the past 2 years. Magnetic resonance imaging revealed a dumbbell shaped and well-outlined highly reflective mass, with its upperpart infiltrating the interspace of the atlanto-occipital joint. The mass was surgically removed. On macroscopic examination, the mass was oblong and partly encapsulated, the size of the mass was 4.9×3.5×3cm, and its cut side was grey. On histologic examination, it showed a disordered collection of bundles of mature striated muscle fibres arranged in a haphazard manner and interspersed with adipose tissue, fibrocytes or mesenchymocytes and collagen, and had a myxoid matrix. On immunochemical examination, mature striated muscle was positive for desmin and myoglobin, adipose tissue and nerves were positive for S-100 protein, and fibrocytes or mesenchymocytes and collagen were positive for vimentin and cluster of differentiation 34. A diagnosis of rhabdomyomatous mesenchymal hamartoma was established.

**Conclusions:**

Rhabdomyomatous mesenchymal hamartoma is a rare dermal or subcutaneous lesion, and we describe its immunohistochemical expression for the first time. This case report provides more information on the microscopic appearance and immunohistochemical expression

## Introduction

Rhabdomyomatous mesenchymal hamartoma (RMH) is a rare dermal or subcutaneous lesion comprising disordered mature adipose tissue, skeletal muscle, adnexal elements, nerve bundles and collagen. Most cases have been described in young patients on their head and neck [1]. We describe a RMH arising in a 31-year-old Chinese man. The lesion occurred on the left side of his neck and under his mastoid process. The size of the lesion was 4.9×3.5×3cm, which was larger than other RMH reported in the English language literature. We describe its immunohistochemical expression for the first time.

## Case presentation

**Figure 1:**
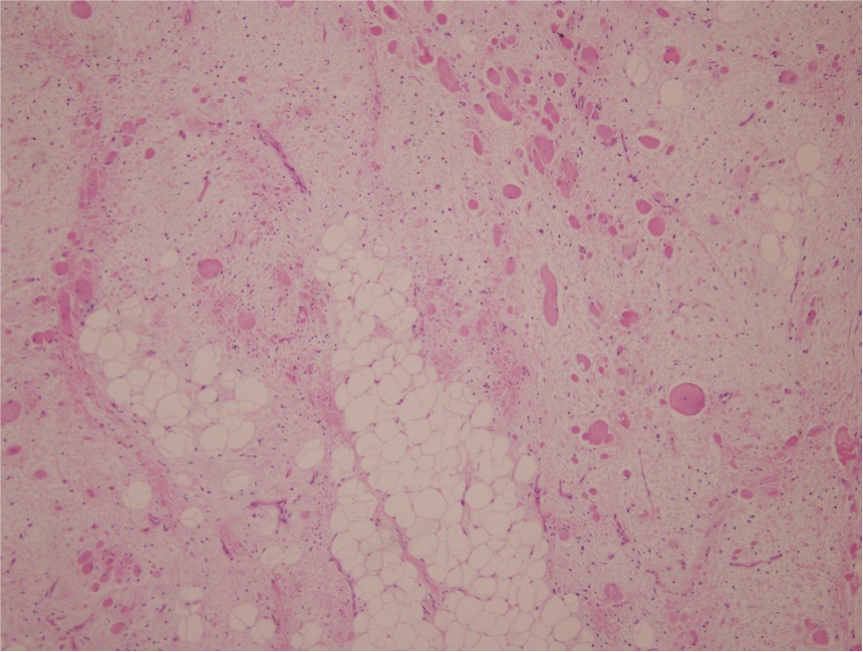
**The mass was composed of disordered skeletal muscle and infiltrated the adipose tissue, fibrocytes or mesenchymocytes and collagen (hematoxylin and eosin stain, original magnification ×10).** Adipose tissue and fibrocytes or mesenchymocytes were admixed with the muscle tissue in varying amounts throughout the lesion.

**Figure 2:**
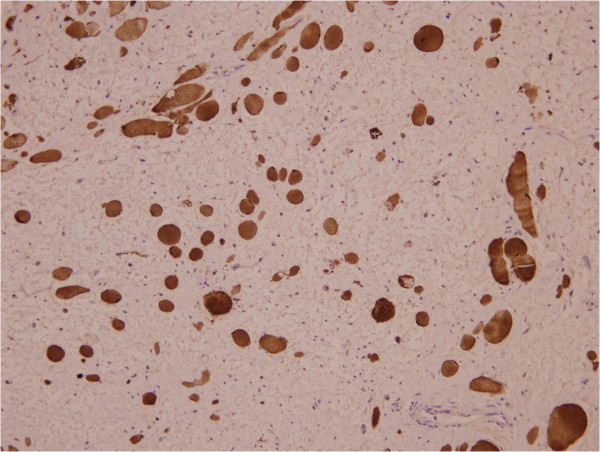
**Immunohistochemistry: the mature striated muscle was positive for myoglobin (Elivision×20).**

**Figure 3:**
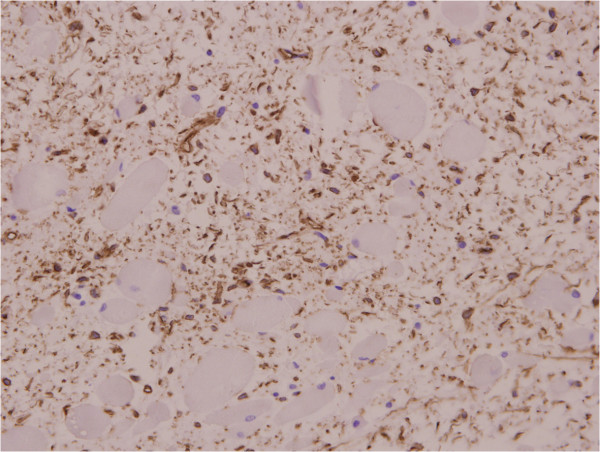
**Immunohistochemistry: the fibrocytes or mesenchymocytes were positive for cluster of differentiation 34 (Envision ×20).**

A 31-year-old Chinese man first presented 2 years previously with a solitary and painless subcutaneous mass on the left side of his neck and under his mastoid process; the size of the mass was 2×2cm. The mass had increased in size in the past 2 years. By the time he saw a doctor, the size of the mass was approximately 5×3cm. The mass was tough to the touch. Ultrasound features included a solid, well-outlined highly reflective mass. Magnetic resonance imaging revealed a dumbbell shaped and well-outlined highly reflective mass, with its upperpart infiltrating the interspace of the atlanto-occipital joint. The initial differential diagnoses included mesenchymal hamartoma, myolipoma, and neural tumour. The mass was surgically removed. During surgery, the tumour felt firm and solid, and it was found to be completely encapsulated. On macroscopic examination, the mass was oblong and partly encapsulated, the size of the mass was 4.9×3.5×3cm, its cut side was grey, and it felt firm. On histologic examination, it showed a disordered collection of bundles of mature striated muscle fibres arranged in a haphazard manner and interspersed with adipose tissue, fibrocytes or mesenchymocytes and collagen, and had a myxoid matrix, mitotic figures were typically rare or absent altogether (Figure 1). On immunochemical examination, mature striated muscle was positive for desmin and myoglobin (Figure 2), adipose tissue and nerves were positive for S-100 protein, and fibrocytes or mesenchymocytes and collagen were positive for vimentin and cluster of differentiation 34 (CD34; Figure 3). The clinical, macroscopic, histologic, and immunochemical characteristics allowed diagnosis of RMH.

## Discussion

RMH was first described as striated muscle hamartoma in 1986 by Hendrick *et al*. [2]. Mills [3] first used the term RMH in 1989 to describe this benign hamartoma. Since 1986, more than 30 cases of RMH have been recognized and reported in the literature. This entity exists under various names including striated muscle hamartoma, congenital midline hamartoma, and hamartoma of cutaneous adnexa and mesenchyme.

RMH is a rare dermal or subcutaneous lesion comprising disordered mature adipose tissue, skeletal muscle, adnexal elements, nerve bundles and collagen [4]. There is no evidence of cellular or malignant degeneration. RMH occurs most commonly in areas where there is superficial striated muscle, such as the nose or chin, followed by the periorbital and anterior neck areas. Most cases have been described in young patients on the head and neck [1], such as periorbital [5], nasal alae [6], lip [7], chin [8], tongue and tonsil [9], and sternoclavicular area [10]. There is no apparent sex predilection. On clinical examination, most RMH are solitary, with a few patients having multiple lesions. Most lesions present as a nodule, papule, skin tag or mass; there was a case presenting as a depressed skin lesion [11]. On macroscopic examination, the size of a lesion varied from 0.3cm to 1.4cm in the reported literature.

In our case the histology was similar to that described for RMH; however the lesion’s size was 4.9×3.5×3cm, was bigger than any other reported cases. To our knowledge, this is the biggest lesion of RMH reported. The mass was partly encapsulated. On histological examination, RMH comprises disordered mature adipose tissue, skeletal muscle, nerve bundles and collagen. The reported cases did not describe the immunohistochemical expression of RMH. In this case, skeletal muscle was positive for desmin and myoglobin, adipose tissue and nerves were positive for S-100 protein, fibrocytes or mesenchymocytes and collagen were positive for vimentin and CD34. It is a new find that the lesion’s stromata were positive for CD34.

The aetiology of RMH is unknown, possible explanations include aberrancy in the embryonic migration of mesodermally derived tissues or a genetic defect predisposing to the formation of hamartomas. Although an association with congenital abnormalities is uncommon, this possibility should be assessed by the clinician.

These lesions are benign, and are typically only excised if causing mass-type effects or for cosmetic reasons.

## Conclusions

RMH is a rare dermal or subcutaneous lesion, and we describe its immunohistochemical expression for the first time. This case report provides more information on the microscopic appearance and immunohistochemical expression of RMH.

## Consent

Written informed consent was obtained from the patient for publication of this case report and any accompanying images. A copy of the written consent is available for review by the Editor-in-Chief of this journal.

### Level of interest

The article is important in the clinical pathology field.

## Abbreviations

CD34: Cluster of differentiation 34
RMH: Rhabdomyomatous mesenchymal hamartoma.
